# Treatment of Adults With Treatment-Resistant Depression: Electroconvulsive Therapy Plus Antidepressant or Electroconvulsive Therapy Alone? Evidence From an Indirect Comparison Meta-Analysis

**DOI:** 10.1097/MD.0000000000001052

**Published:** 2015-07-02

**Authors:** Guo-Min Song, Xu Tian, Ting Shuai, Li-Juan Yi, Zi Zeng, Shuang Liu, Jian-Guo Zhou, Yan Wang

**Affiliations:** From Department of Nursing, Tianjin Hospital, Tianjin, Peoples’ Republic of China (G-MS); Graduate College, Tianjin University of Traditional Chinese Medicine, Tianjin, Peoples’ Republic of China (XT, TS, L-JY, ZZ); School of Nursing, Tianjin University of Traditional Chinese Medicine, Tianjin, Peoples’ Republic of China (XT, TS, L-JY, ZZ, YW); School of Nursing, Peking Union Medical College, Beijing, Peoples’ Republic of China (SL); and Department of Oncology, Affiliated Hospital of Zunyi Medical College, Zunyi, Peoples’ Republic of China (J-GP).

## Abstract

Electroconvulsive therapy (ECT) and antidepressant are the effective treatment alternatives for patients with treatment-resistant depression (TRD); however, the effects and safety of the ECT plus antidepressant relative to ECT alone remain controversial. We decide to assess the potential of ECT plus antidepressant compared with ECT alone by undertaking an indirect comparison meta-analysis.

Databases from PubMed, ISI Web of Science, CENTRAL, Clinicaltrials.gov, EMBASE, CBM (China Biomediccal Literatures Database), and CNKI (China National Knowledge Infrastructure) were searched for relevant studies through November 21, 2014. Literature was screened, data were extracted and methodological quality of the eligible trial was assessed by 2 independent reviewers accordingly. Then, head-to-head and indirect comparison meta-analyses were carried out.

A total of 17 studies which including 13 studies regarding ECT plus antidepressant versus antidepressant alone and 4 studies concerning ECT versus antidepressant alone containing a total of 1098 patients were incorporated into this meta-analysis. The head-to-head comparison suggested that response rate can be improved in the ECT plus antidepressant (RR, 1.82; 95% CI, 1.55–2.14) and ECT alone group (RR, 2.24, 95% CI, 1.51–3.33) compared with antidepressant alone, respectively; adverse complications including memory deterioration and somatization were not significantly increased except incidence of memory deterioration in ECT plus antidepressant in the 4th weeks after treatment (RR, 0.09, 95% CI, 0.02–0.49). Indirect comparison meta-analysis showed that no significant differences were detected in response rate and memory deterioration between ECT plus antidepressant and ECT alone. However, ECT plus antidepressant increased the incidence of memory deterioration relative to ECT alone.

With present evidence, the regime of ECT plus antidepressant should not be preferentially recommended to treat the patients with TRD relative to ECT alone.

## INTRODUCTION

Depression is a condition characterized by poor response and prognosis, as well as, it is associated with lower quality of life of patients and higher mortality.^1^ It is predicted that depression will be listed as the second highest cause to result in huge economic burden by 2020.^2^ Published evidences suggested that approximately 30% of patients with depression do not response to treatment with at least a tricyclic antidepressant (TCA) at a minimum dose of 150 mg/day of imipramine (or equivalent drug) for 4 to 6 weeks’, which condition was defined as treatment-resistant depression (TRD).^3,4^

Treatment for TRD has been becoming a thorny problem through a diversity of treatment modalities has been developed. Previously published randomized controlled trials (RCTs) and systematic reviews revealed that antidepressant, especially selective serotonin reuptake inhibitors (SSRIs), may be a potential agent to improve the status of adults with TRD,^5–7^ whereas, a series of serious adverse reactions limit the use of antidepressant.^8^ To address the issues caused by the use of antidepressant, electroconvulsive therapy (ECT), which is commonly recognized as an effective therapeutic intervention targeted at patients with TRD, was developed.^9^ Previous trials suggested that 80–90% of patients with depression showed a marked effective response to ECT alone.^10^ However, side effects which mainly included deterioration, epilepsy unspecified, headache, confusion of consciousness, and fracture of ECT hinder its application, just like antidepressant.^11^ Consequently, some studies explored the potential of ECT plus antidepressant for treating patients with TRD.^12–14^ However, the effects and safety of ECT plus antidepressant for the treatment of patients with TRD relative to ECT alone was inconclusive due to no direct comparison of both was performed before.

To resolve the issues, therefore, we undertook this head-to-head and indirect comparison meta-analysis to evaluate the effects and safety of ECT combined antidepressant compared with ECT alone for the treatment of TRD.

## MATERIALS AND METHODS

The Preferred Reporting Items for Systematic Reviews and Meta-analysis (PRISMA) statement^15^ and Cochrane Handbook for Systematic Reviews of Interventions are adopted to as the guideline of planning the systematic review and meta-analysis.^16^ All pooled analyses are based on previously published studies, and thus no ethical approval and patient consent are required.

### Search Strategy and Selection Criteria

Electronic search of PubMed, ISI Web of Science, CENTRAL (the Cochrane Central of Registration Controlled Trials), Clinicaltrials.gov, EMBASE, SinoMed (China Biomedical Literatures Database), and CNKI (China National Knowledge Infrastructure) was performed for RCTs concerning the effects and safety of ECT combined antidepressant relative to antidepressant alone and ECT alone compared with antidepressant alone through November 2014 by using the combination of text words and medical subject heading terms (MeSH). The electronic search combined the terms related to treatment-resistant depression (including “depressive disorder, treatment-resistant,” “TRD,” “disorder∗, treatment-resistant depressive,” “treatment-resistant depressive disorder∗,” “therapy-resistant depression∗,” “depression∗, therapy-resistant,” “therapy resistant depression∗,” “treatment-resistant depression∗,” “depression∗, treatment resistant,” “resistant depression∗, treatment,” “refractory depression∗,” “depression∗, refractory,” “intractable depression∗’), terms related to electroconvulsive therapy (including “electroconvulsive therap∗,” “therap∗, electroconvulsive,” “ECT,” “psychotherap∗,” “shock therap∗, electric,” “electric shock therap∗,” “therap∗, electric shock,” “convulsive therap∗, electric,” “electric convulsive therap∗,” “therap∗, electric convulsive,” “therap∗, electroshock’) and terms related to random (including “randomized controlled trial,” “randomized controlled trials as topic,” “controlled clinical trial,” “controlled clinical trial as topic,” “random∗’). The detailed search strings for PubMed, CENTRAL and EMBASE were summarized in an additional DOC file (Additional File 1). The reference lists of eligible studies and relevant reviews were manually checked for including any relevant articles. Two independent reviewers (XT and TS) critically checked citations identified by 2 steps of reading the titles/abstracts and full-texts.

According to *PICOS* acronym, we identified following selection criteria: Participants (***P***): adult patients were diagnosed as TRD; Intervention (***I***): ECT plus antidepressant/ECT alone; Comparison (***C***): Antidepressant alone; Outcomes (***O***): response rate and adverse reactions including memory deterioration and somatization; and Study design (***S***): RCT.

We will exclude these studies if met one of following criteria: animal study and experiment; the essential information was not available to extract the data and cannot acquire original data via contacting authors; for republishing studies or that was the same study from different follow time and research department; and nonoriginal research, such as review, letter, etc.

### Data Extraction and Outcome Measures

Data were independently extracted by reviewers (G-MS and XT) using the predesigned table (Additional File 2), which included first author, publication year, sample size (Male/Female), length of illness, duration of treatment, interventions (Study group/Control group), diagnosis criteria of TRD, study design, the interesting outcome measures. Authors were contacted in case of essential data were not available. All information will be rechecked by a third author (L-JY). Any discrepancies were resolved by consensus.

The estimates of outcome measures including response rate and side effects including memory deterioration and somatization symptom, in this study, were calculated to assess the potential of ECT combined with antidepressant versus ECT alone. Recovery, remission, and determining response were defined as a 75%, 50%, and 25% HAMD score reduction from baseline by the end of treatment, respectively, and then divide overall number of patients by number of patients who have diagnosed as recovery, remission, and determining response, and we can get response rate.^8,12,13^ No instrument was described in published articles to assess the memory deterioration and only events were reported, then we performed pooled analysis according to the data presented in the original study. The somatization symptom resulted from some psychological problems caused by TRD such as mental conflict and ambivalent refer to headache, dizzy, nausea, and insomnia, etc.

### Assessing the Risk of Bias

Two independent reviewers (ZZ and YW) assessed the risk of bias of studies included in accordance with the Cochrane Collaboration's tool for risk of bias assessment.^16^ Evaluation index included selection bias, performance bias, attrition bias, detection bias, reporting bias, and other potential source of bias. According to the information extracted from primary studies, each domain was rated as “high risk,” “unclear risk,” or “low risk.” The overall risk of bias of a study was concluded by summarizing all the 6 aspects. The summary risk of bias was considered to be low which corresponding A grade (low risk in all domains), unclear which corresponding B grade (unclear risk in 1 or more domains), or high which corresponding C grade (high risk in 1 or more domains). Any disagreements were resolved by consultation of a third reviewer (J-GZ and SL) or based on consensus.

### Statistical Analysis

Indirect comparison may be an alternative by using common comparator under the given condition, in which no head-to-head comparison to evaluate the potential of different interventions.^16–20^ For head-to-head comparison on the topic of ECT plus antidepressant versus antidepressant alone and ECT versus antidepressant alone, the classic meta-analysis will be performed to obtain corresponding estimates of effect.

At the stage of classic meta-analysis, all outcomes were reported in relative risk (RR) with 95% confidence intervals (CIs). We extracted end-point data to calculate the pooled effect size due to baselines between study and control groups was consistent for each study included in this meta-analysis.

We adopted fixed- or random-effects model based on Mantel–Haenszel (M-H) or inverse variance (I-V) statistical approach to perform corresponding meta-analyses according to the characteristics of clinical and methodology. A fixed-effects model was selected if no significant difference existed in those studies which included into the given outcome measure regardless of level of heterogeneity, in contrast, a random-effects model was selected. The inconsistency across studies was tested by using the I^2^ statistic and Cochrane Q. I^2^ statistic represents the proportion of variation on account of heterogeneity instead of chance and is perceived to be low (25% ≤I^2^ ≥ 50%), moderate (50% ≤I^2^ 75%), and high (I^2^ ≥ 75%).^16^ I^2^ ≥ 50% and Q test with *P* < 0.10 suggested a significantly high heterogeneity. To reduce the likelihood of spurious results, subgroup analysis according to the duration of treatment was prespecified to evaluate their impact on the pooled estimate and heterogeneity. We performed sensitivity analysis by excluding study of low quality and studies that were significantly different from others for testing the robust of pooled results. A 2-sided *P* < 0.05 was regarded as statistical significance except where it was emphasized particularly. Based on the estimates of direct comparison, estimating differences in effects and safety of ECT plus antidepressant and ECT alone according to the function were performed.^19^

All extracted data were introduced in Stata 12.0 software (StataCorp, College Station, TX) for statistical analysis and the risk of bias was assessed by using RevMan version 5.3 (The Cochrane Collaboration, Software Update, Oxford, UK). Owing to the limited number (below 10) of studies included in each analysis, publication bias was not assessed.

## RESULTS

### Study Identification and Selection

**FIGURE 1:**
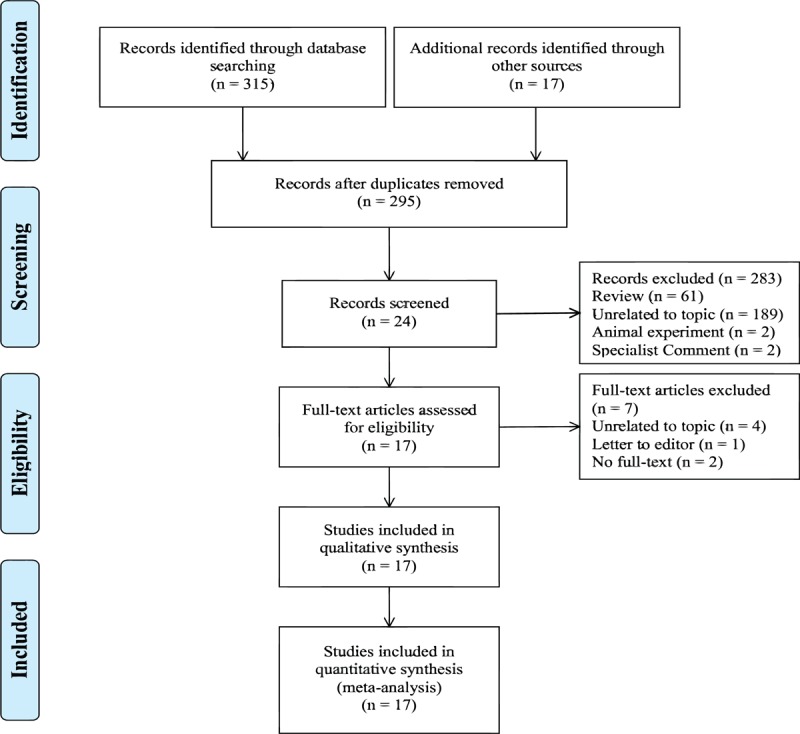
Flow chart of citations retrieval and selection.

We identified 322 citations in the initial database search and other sources stage. Of them, 27 were duplicated by using EndNote 7.1 software, and 271 were excluded after screened titles and abstracts according to the selection criteria. After a detailed assessment of the remaining 24, 17 were excluded result from unrelated to given topic, letter to the editor, and no full-text (in Polish). Consequently, 17^10,12–14,21–33^ which including 13 studies^12–14,24–33^ regarding ECT plus antidepressant versus antidepressant alone and forth studies^10,21–23^ concerning ECT compared with antidepressant alone, eligible studies were applicable for meta-analysis. Flow chart of the study retrieval and selection is presented in Figure 1.

### Study Characteristics

The characteristics of each study included into this meta-analysis are presented in Table 1  . These studies were published spanning from 1997 to 2014, but most of them published in a range of 2010 to 2014.^12–14,21–23,25,26,28–30,32^ For all eligible studies, 16 of them were conducted in China except for 1 was conducted in Germany.^10^ Sample size of each eligible study incorporated into our study varied from 21 to 100 and in total of 1098 patients. Duration of treatment was at least 4 weeks and 2 studies lasted for 6 weeks,^13,28^ as well as, 4 studies^12,14,21,26^ were stopped in the 8th weeks after treatment. Length of illness of patients enrolled have obvious difference, which varied from a few months to several years and 2 studies did not report details.^22,23^ Antidepressant, which regarded as common comparator, included Paroxetine, Clomipramine, Citalopram, Sertraline, Venlafaxine, Amitriptyline, Fluvoxamine, Fluoxetine, and Mirtazapine, in addition, 3 studies^23,24,32^ did not provide details of medications just stated that antidepressant were prescribed in accordance with standard criteria. We summarized the details of receptors and pharmacokinetic of different antidepressants listed in our study according to references,^34–37^ and all information are presented in Table 2.

**TABLE 1:**
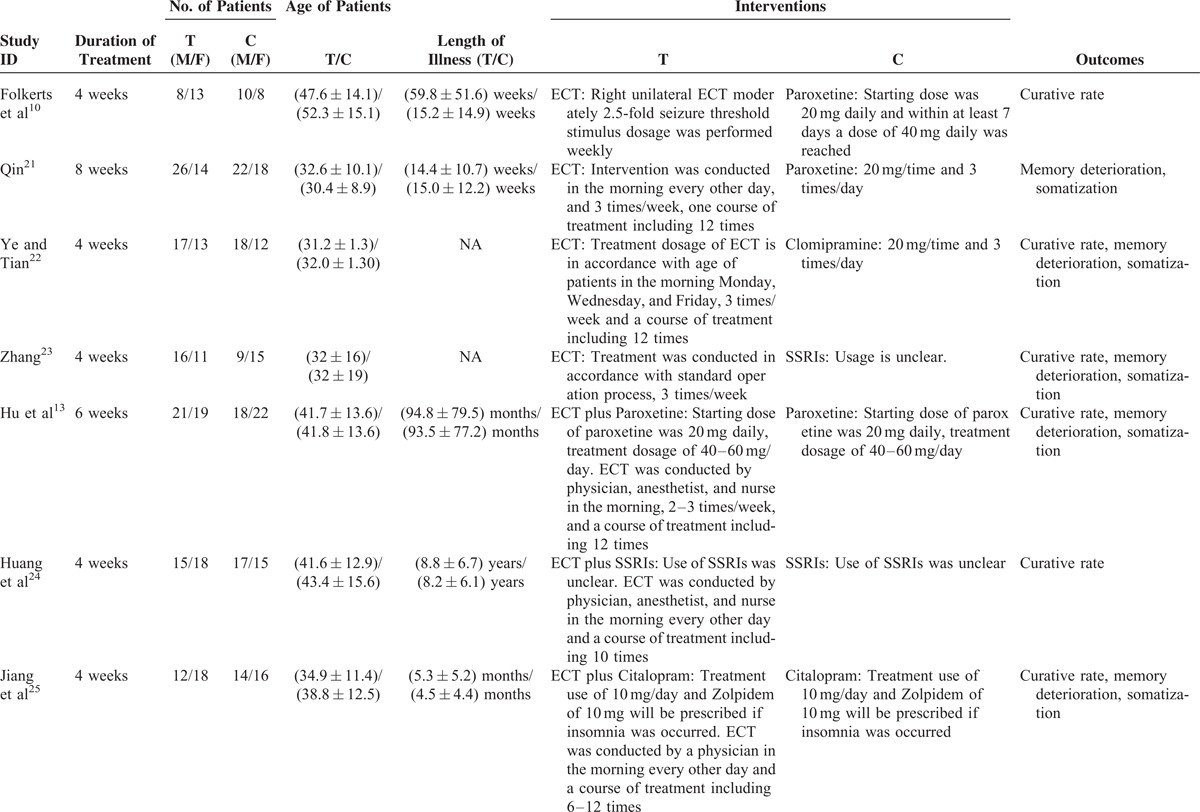
Characteristics of Each Study Included Into this Meta-Analysis

**TABLE 1 (Continued):**
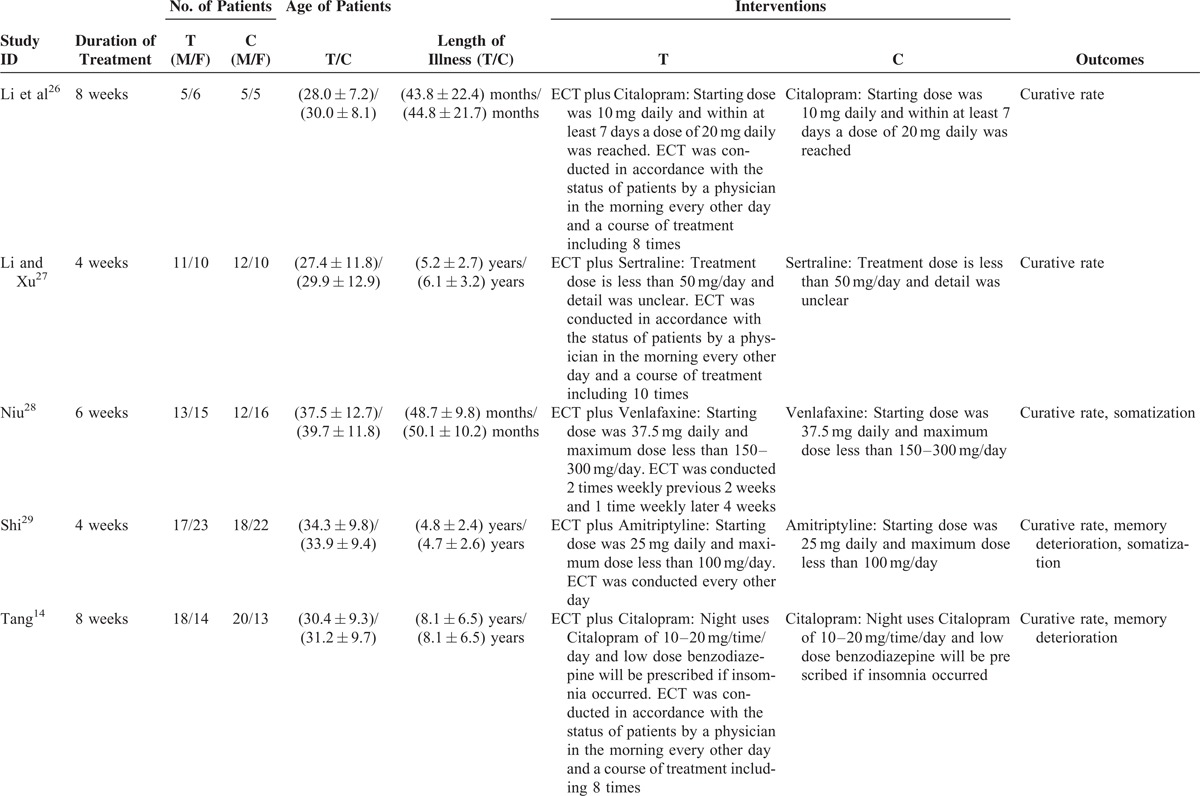
Characteristics of Each Study Included Into this Meta-Analysis

**TABLE 1 (Continued):**
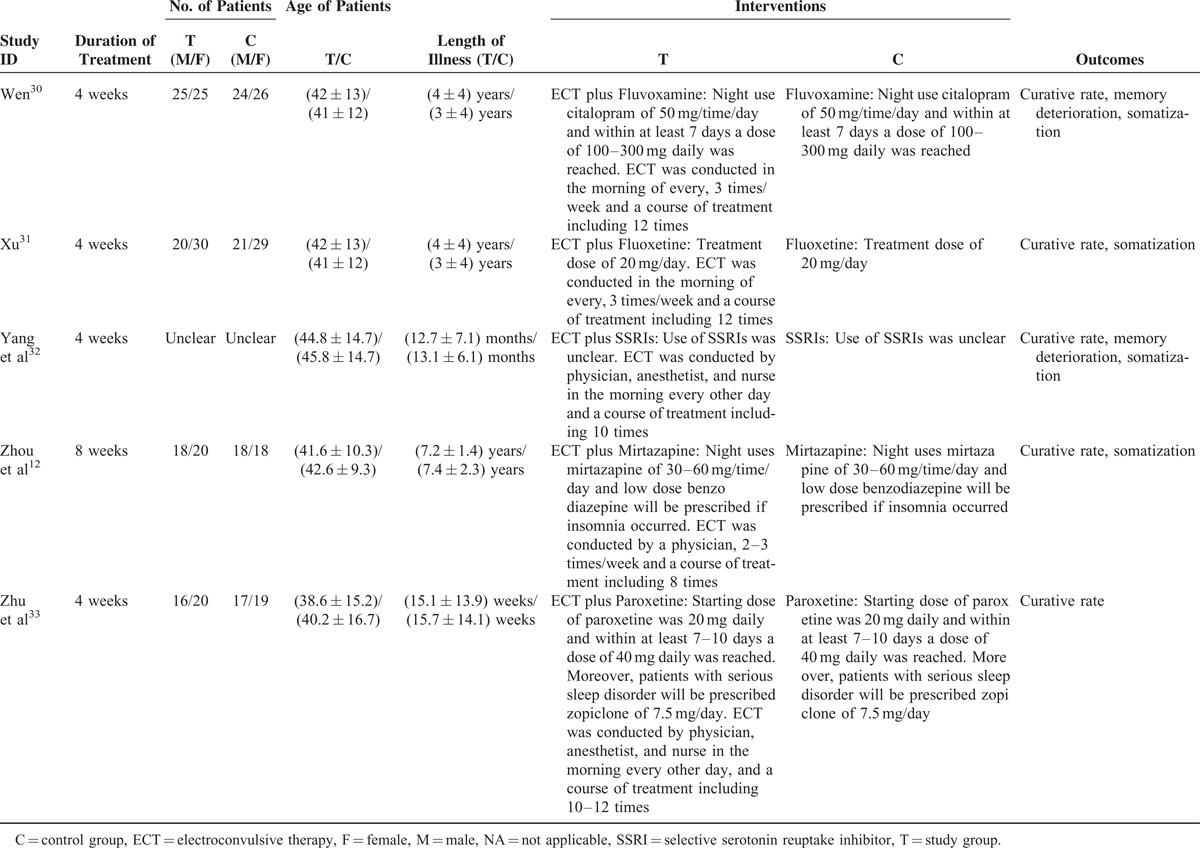
Characteristics of Each Study Included Into this Meta-Analysis

**TABLE 2:**
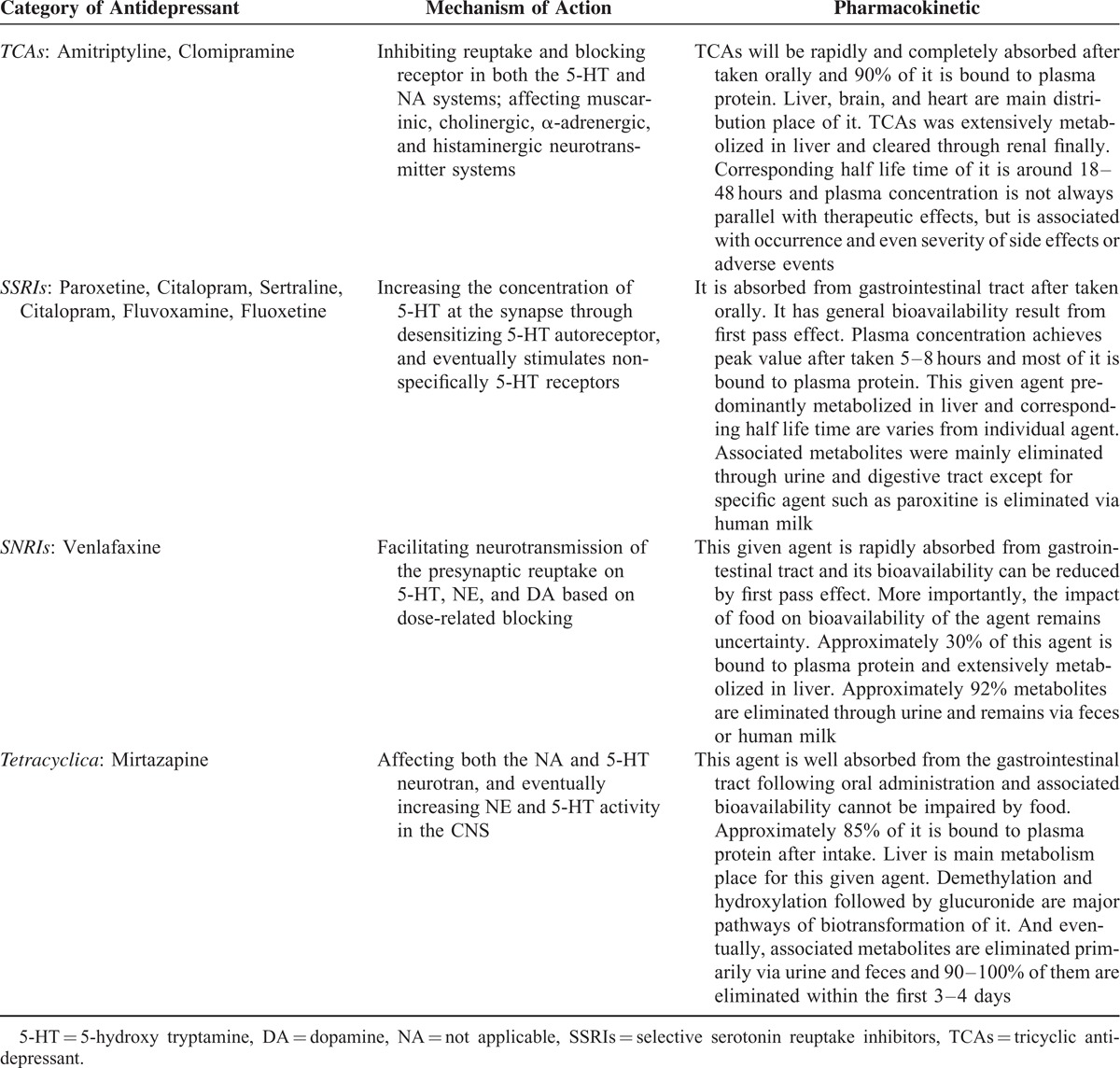
Receptors and Pharmacokinetic of Different Antidepressants Listed in Our Study

### Assessing Risk of Bias

**FIGURE 2:**
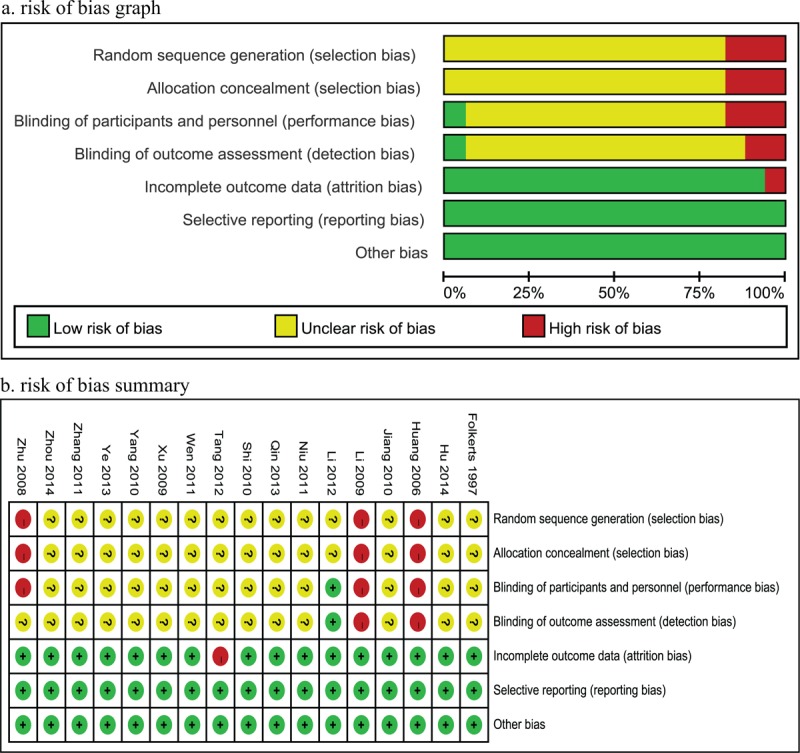
Assessment of risk of bias.

A total of 17 eligible studies were incorporated into the meta-analysis. Three^24,27,33^ of these trials have selection bias, performance bias and detection bias. Only 1^26^ performed appropriate blinding method to avoid performance and detection bias. One study^14^ did not perform intention-to-treat (ITT) analysis to deal drop-outs and other potential bias resources did not exist in all trials. According to the assessment of risk of bias for each study, no study was classified into grade A for overall quality of methodology, 13 studies were rated as B grade and 4 studies were rated as C. Assessing risk of bias outcome is shown in Figure 2.

### Response Rate

#### Meta-Analysis on ECT Plus Antidepressant Versus Antidepressant Alone

**FIGURE 3:**
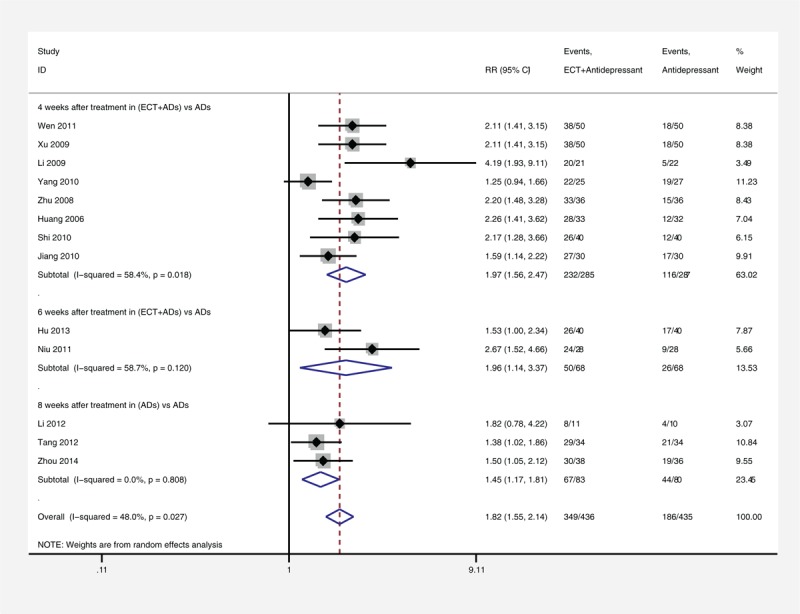
Meta-analysis on response rate of ECT plus antidepressant versus antidepressant alone.

Thirteen studies^12–14,24–33^ which investigated the response rate of ECT combined with antidepressant relative to antidepressant alone were included into this meta-analysis to obtain an estimate. Eight^24,25,27,29–33^ of them were completed in duration of treatment of 4th weeks, 2^13,29^ lasted 6 weeks, and 3^12,14,26^ obtained data lasted for 8 weeks after treatment. A subgroup analysis was adopted for the outcome according to different duration of treatment. We identified that the length of illness and age of patients for study performed by Li and Xu^27^ and Yang et al^32^ was different from remained, however, no obvious different existed in the studies completed in the 6th weeks after treatment, in addition, I^2^ of 58.4% with *P* of 0.02 and I^2^ of 58.7% with *P* of 0.12 was estimated in the 4th and 6th weeks after treatment, consequently, we adopted a random-effects model based on the M-H method to perform the pooled analysis. Meta-analysis suggested that ECT plus antidepressant can effectively increase the response rate relative to antidepressant alone (RR, 1.82; 95% CI, 1.55–2.14), the pooled result is presented in Figure 3.

#### Meta-Analysis on ECT Versus Antidepressant Alone

**FIGURE 4:**
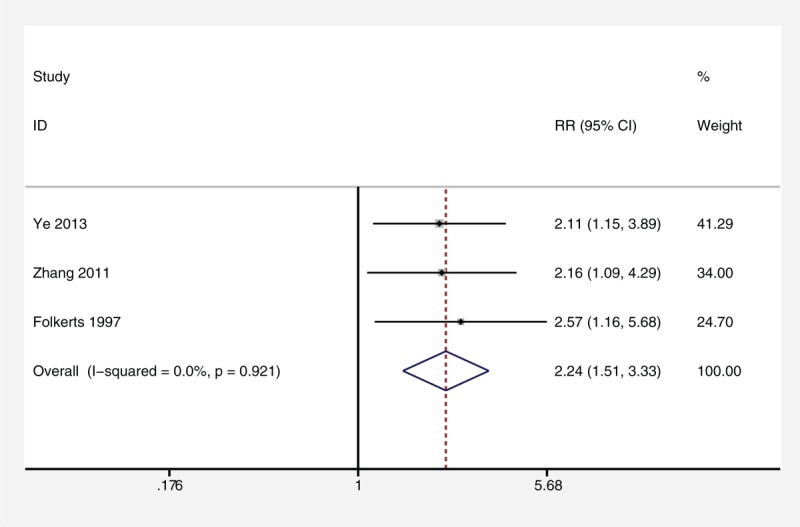
Meta-analysis on response rate of ECT versus antidepressant alone.

Three eligible studies^10,22,23^ reported the outcome measures of response rate and all incorporated into the meta-analysis to summarize the evaluation. All studies included lasted for 4 weeks and no special analysis was carried out. The clinical characteristics and methodology were considered to be as homogeneity and the heterogeneity test indicated low variance across studies (I^2^ = 0.0%, *P* = 0.92). And then, a fixed-effects model was adopted. Meta-analysis showed that ECT can effectively attenuate symptoms of patients with TRD compared with antidepressant alone (RR, 2.24; 95% CI, 1.51–3.33), the summarized estimate is shown in Figure 4.

#### Indirect Comparison Meta-Analysis on ECT Plus Antidepressant Versus ECT Alone

To perform an indirect comparison meta-analysis to assess the potential of ECT plus antidepressant versus ECT alone, we analyzed the clinical characteristics and methodology of this trials included into direct comparison meta-analysis and in-transitivity was not be detected. Hitherto, 8 eligible studies^24,25,27,29–33^ in ECT combined with antidepressant versus antidepressant group and 3^10,22,23^ in ECT versus antidepressant group were selected to conduct an indirect comparison meta-analysis on curative rate based on duration of treatment of 4 weeks. The result suggested that no significant difference was detected between ECT plus antidepressant and ECT alone (RR, 0.81; 95% CI, 0.52–1.52).

### Memory Deterioration

#### Meta-Analysis on ECT Plus Antidepressant Versus Antidepressant Alone

**FIGURE 5:**
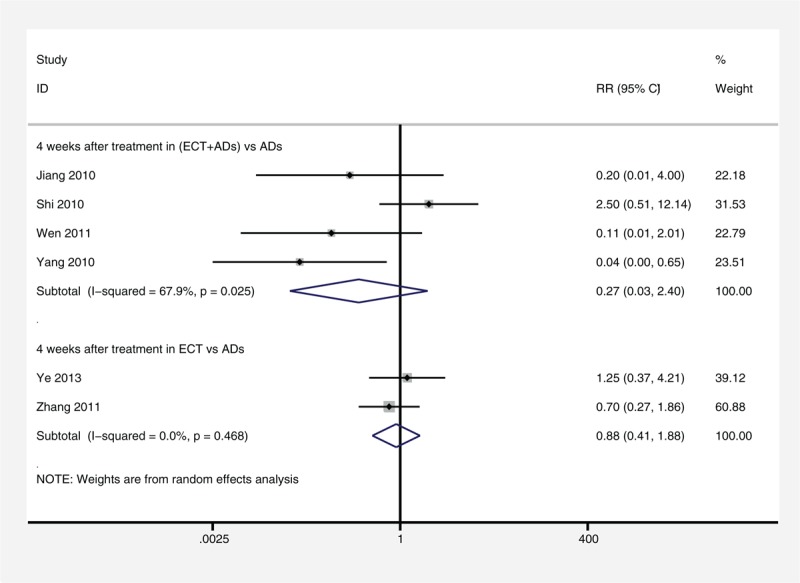
Meta-analysis on memory deterioration of ECT plus antidepressant vs. antidepressant alone.

Four studies^25,29,30,32^ concerning the memory deterioration between ECT plus antidepressant and antidepressant alone were enrolled into this meta-analysis. No significant difference existed in these studies though the I^2^ of 67.9% with *P* of 0.03 was calculated. So we selected a random-effects model to perform the meta-analysis. The pooled result revealed that ETC combined with antidepressant may be not associated with a higher rate of memory deterioration (RR, 0.27; 95% CI, 0.03–2.40), the result is presented in Figure 5.

#### Meta-Analysis on ECT Versus Antidepressant Alone

Two studies^22,23^ reported the events of memory deterioration and thus were included into this meta-analysis. These eligible studies were considered to be as homogeneity and variance test indicated an I^2^ of 0.0% with *P* of 0.47. Consequently, the fixed-effects model was adopted. The meta-analysis showed that ECT will not cause more memory deterioration compared with antidepressant alone (RR, 0.88; 95% CI, 0.41–1.88), the result is presented in Figure 5.

#### Indirect Comparison Meta-Analysis on ECT Plus Antidepressant Versus ECT Alone

Similarity was found after assessed the characteristics of participants, interventions, outcome measures, methodology, and so on. Based on the length of treatment, 4 studies^25,29,30,32^ regarding events of memory deterioration in ECT plus antidepressant versus antidepressant alone and 2^22,23^ concerning that of in ECT versus antidepressant alone were included into this indirect comparison meta-analysis to obtain the evaluation of events of memory deterioration. The result suggested that no significant difference was identified between ECT plus antidepressant and ECT alone (RR, 0.60; 95% CI, 0.15–2.36).

### Somatization

#### Meta-Analysis on ECT Plus Antidepressant Versus Antidepressant Alone

**FIGURE 6:**
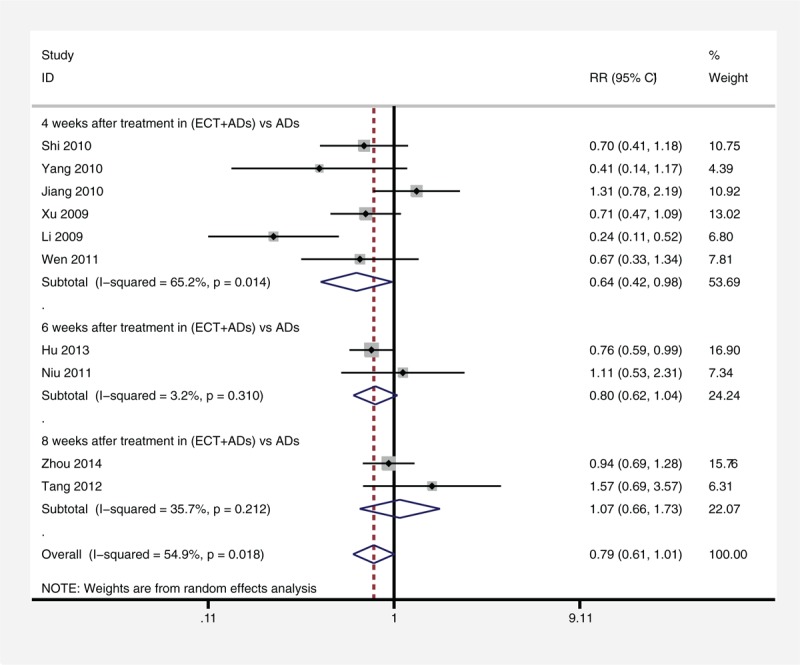
Meta-analysis on somatization of ECT plus antidepressant versus antidepressant alone.

Ten eligible studies^12–14,25,27–32^ reported the incidence of somatization and thus were all incorporated into the meta-analysis. We identified that the length of illness and age of patients for study performed by Li and Xu^27^ and Yang et al^32^ was different from remained, however, no obvious different existed in the studies completed in the 6th weeks after treatment, in addition, I^2^ of 65.2% with *P* of 0.01 and I^2^ of 54.9% with *P* of 0.02 was estimated in the 4th and 8th weeks after treatment, consequently, we adopted a random-effects model based on the M-H method to perform the pooled analysis. Meta-analysis suggested that ECT plus antidepressant increased the incidence of somatization of patients with TRD compared with antidepressant in the 4th weeks after treatment (RR, 0.64; 95% CI, 0.42–0.98), but the incidences were not significant difference in the 6th and 8th weeks after treatment, the result is presented in Figure 6.

#### Meta-Analysis on ECT Versus Antidepressant Alone

**FIGURE 7:**
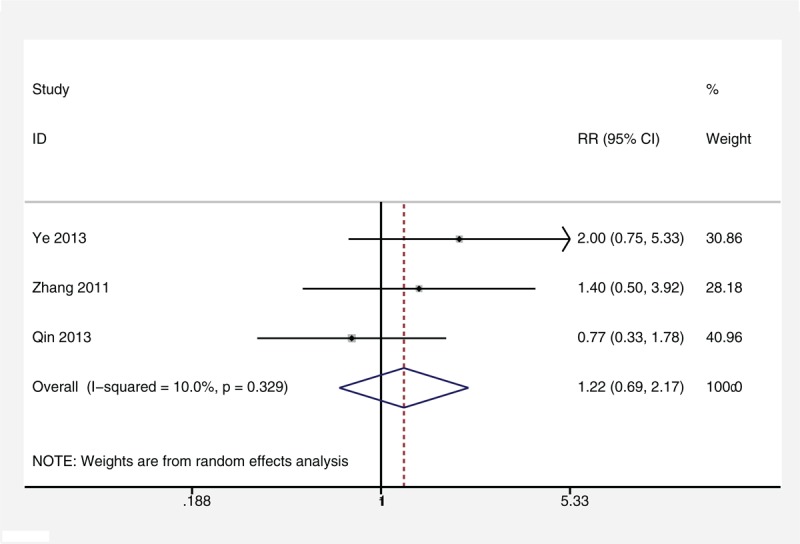
Meta-analysis on somatization of ECT versus antidepressant alone.

Data of somatization can be extracted from 3 studies^21–23^ in ECT versus antidepressant alone group and were all included into this meta-analysis. No significant variance was identified across studies and variance test generated an I^2^ of 10.0% with *P* of 0.33, and thus a fixed-effects model was selected. Meta-analysis showed that no significant difference was detected between ECT compared with antidepressant alone (RR, 1.22; 95% CI, 0.69–2.17), the result is presented in Figure 7.

#### Indirect Comparison Meta-Analysis on ECT Plus Antidepressant Versus ECT Alone

These trials have transitivity in terms of clinical characteristics and methodology and this condition met the criteria to perform an indirect comparison meta-analysis. Six studies^25,27,29–32^ in ECT plus antidepressant versus antidepressant and 3^21–23^ in ECT versus antidepressant regarding incidence of somatization in 4th weeks after treatment was all incorporated into this indirect comparison meta-analysis to obtain an evaluation. Meta-analysis suggested that no significant difference was identified between ECT combined with antidepressant and ECT alone (RR, 0.58; 95% CI, 0.05–7.29).

### Sensitivity Analysis

**FIGURE 8:**
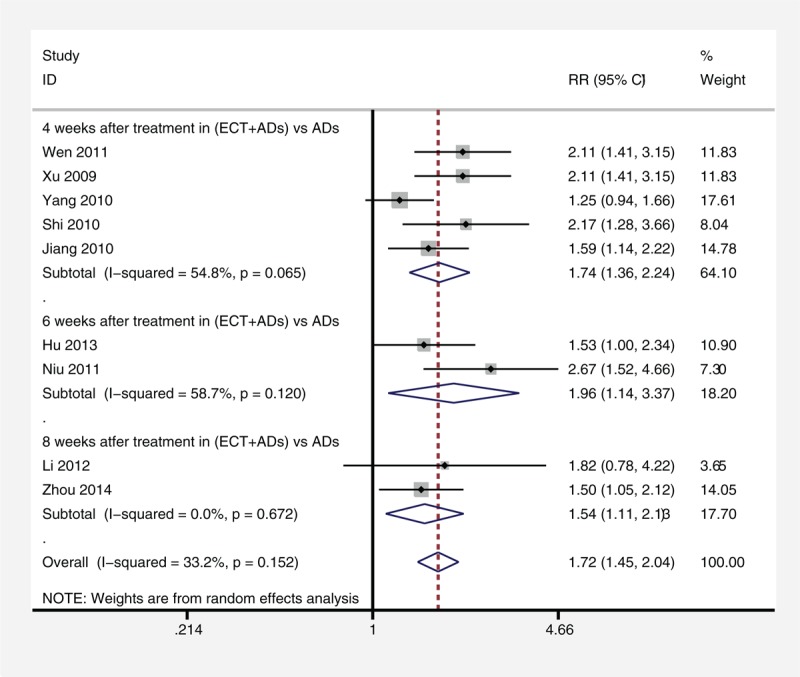
Sensitivity analysis on response rate by excluding study of low quality.

**FIGURE 9:**
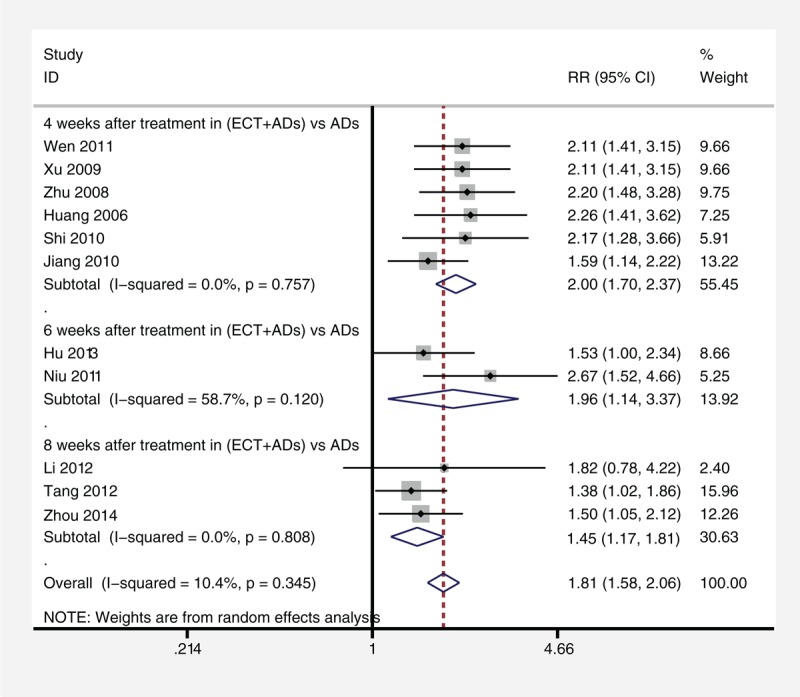
Sensitivity analysis on response rate by excluding heterogeneous study.

A sensitivity analysis was conducted by excluding study of low quality and studies that were significantly different from others. Four eligible studies were rated as grade C in quality of methodology. For curative rate, 3 studies and one that rated as low quality fallen into the 4th and 8th weeks after treatment, respectively, and the sensitivity analysis suggested that the pooled results were robust (Figure 8). Moreover, for the same outcome, study performed by Li and Xu^27^ and Yang et al^32^ was different from remained, consequently, the sensitivity analysis was also performed by excluding the 2 studies and result indicated a robust pooled results (Figure 9). For the outcome of memory deterioration, a study planned by Shi may be potentially heterogeneous source that caused the variance, and then we performed a sensitivity analysis to test the robust of pooled results. The analysis validated the statement provided above (I^2^ = 0.0%, *P* = 0.00) and the pooled result showed that ECT combined with antidepressant increased the incidence of memory deterioration (RR, 0.09; 95% CI, 0.02–0.49). For somatization, the study performed by Li et al and Yang et al was potential heterogeneous factors resulted in heterogeneity in the given outcome in the 4th weeks after treatment. So a sensitivity analysis by excluding separate study was carried out. The analysis established the preanalysis (I^2^ = 29.9%, *P* = 0.23) and showed that the incidence of somatization in the 4th weeks after treatment was not significant different in terms of ECT plus antidepressant relative to antidepressant (RR, 0.82; 95% CI, 0.60–1.21).

## DISCUSSION

TRD is still an extremely difficult problem due to lack of effective treatment agents and comprehensive intervention regimes.^3,38,39^ Antidepressant (especially SSRIs) and ECT, at present, play an important role in the treatment of adults with TRD^3,10,40^ and this 2 treatment approaches still extensively prescribed until now. ECT is still considered an effective treatment approach in management of TRD.^10,12–14^

The mechanism of ECT treating TRD is still uncertain.^41^ Many of previous summaries include 2 points: ECT can increase the concentration of prolactine (PRL) transiently and then the levels of dopamine (DA) and 5-hydroxy tryptamine (5-HT) were increased^42^; and the concentration of brain-derived neurotrophic factor (BDNF) was increased under the stimulation by using ECT and then effectively improve the efficacy.^43,44^ Our meta-analysis also validated that ECT combined with antidepressant or ECT alone are effective alternatives compared with antidepressant alone in treating patients with TRD although higher incidence of somatization occurred in the ECT plus antidepressant in the 4th weeks after treatment.

Head-to-head comparison meta-analysis in prospective RCTs is the best approach to answer some questions.^44^ This method, unfortunately, cannot be carried out when lack of direct comparison RCTs on different interventions.^17,19,45^ To evaluate the effects and safety of ECT plus antidepressant relative to ECT alone, hence, we undertake the indirect comparison meta-analysis. To our knowledge, this is the first meta-analysis to systematically evaluate the effects and safety of ECT plus antidepressant versus ECT alone by using indirect comparison. The meta-analysis showed that ECT combined with antidepressant cannot improve the curative rate, decrease the incidence of adverse including memory degeneration and somatization compared with ECT alone. It is very important that ECT combined with antidepressant increased the incidence of memory deterioration of TRD in the 4th weeks after treatment relative to ECT alone, however, this condition was not detected in ECT alone versus antidepressant group.

The studies of low quality and heterogeneous studies included into this meta-analysis, and this condition may reduce the power of meta-analysis. Meanwhile, the sensitivity analysis suggested that studies of low quality and heterogeneous studies negatively affected the pooled results. Consequently, more large-scale and well-designed RCTs are still warranted.

## LIMITATIONS

There exist a number of limitations in this meta-analysis, which need to be acknowledged. Firstly, only a small number of eligible studies were included to assess the potential of ECT versus antidepressant alone, and thus reducing the power of our study. Small sample size is the fatal short for all eligible studies and it may lead to a negative result. At the stage of accessing full-text, 2 articles are possible to be included into this meta-analysis, however, the full-text cannot be obtained due to the condition of no access and therefore, selection bias may reduce the robust of our meta-analysis. Although no language restriction was imposed, some databases indexed in non-English and Chinese were not searched, it also contributed to selection bias. In all of the trials included in the study, no study was classified as grade A and 4 studies were rated as grade C. Inadequate methodology impaired the pooled results also. No definitive instruments for assessed the status of adverse actions including memory deterioration and somatization symptom were described in all eligible studies and the pooled results may be impaired. Finally, the publication bias test was not conducted due to insufficient number of eligible studies for each outcome (subgroup) and thus the pooled results will be negatively affected if small sample size effect existed.

## CONCLUSIONS

There exist insufficient high-quality evidence applicable in the current literature regarding the effectiveness and safety of ECT combined with antidepressant relative to ECT alone for the treatment of patients with TRD. Hence, the findings from this indirect comparison meta-analysis are by no means definitive. Nevertheless, the findings suggested that ECT combined with antidepressant cannot effectively improve the clinical outcomes of patients with TRD compared with ECT alone. In contrast, ECT combined with antidepressant will increase the incidence of memory deterioration relative to ECT alone in the 4th weeks after treatment. In conclusion, the regime of ECT plus antidepressant should not be prior recommended to treat the patients with TRD relative to ECT alone.
